# ASIA: Automated Social Identity Assessment using linguistic style

**DOI:** 10.3758/s13428-020-01511-3

**Published:** 2021-02-11

**Authors:** Miriam Koschate, Elahe Naserian, Luke Dickens, Avelie Stuart, Alessandra Russo, Mark Levine

**Affiliations:** 1 Department of Psychology, University of Exeter, Washington Singer Laboratories, Exeter, EX4 4QG UK; 2 Institute for Data Science and AI, University of Exeter, Exeter, UK; 3 Department of Information Studies, University College London, London, UK; 4 Department of Computing, Imperial College London, London, UK; 5 Department of Psychology, Lancaster University, Lancaster, UK

**Keywords:** Social categorization, Social identity, Natural language processing, Social media data, Psychological assessment

## Abstract

The various group and category memberships that we hold are at the heart of who we are. They have been shown to affect our thoughts, emotions, behavior, and social relations in a variety of social contexts, and have more recently been linked to our mental and physical well-being. Questions remain, however, over the dynamics between different group memberships and the ways in which we cognitively and emotionally acquire these. In particular, current assessment methods are missing that can be applied to naturally occurring data, such as online interactions, to better understand the dynamics and impact of group memberships in naturalistic settings. To provide researchers with a method for assessing specific group memberships of interest, we have developed ASIA (Automated Social Identity Assessment), an analytical protocol that uses linguistic style indicators in text to infer which group membership is salient in a given moment, accompanied by an in-depth open-source Jupyter Notebook tutorial (https://github.com/Identity-lab/Tutorial-on-salient-social-Identity-detection-model). Here, we first discuss the challenges in the study of salient group memberships, and how ASIA can address some of these. We then demonstrate how our analytical protocol can be used to create a method for assessing which of two specific group memberships—parents and feminists—is salient using online forum data, and how the quality (validity) of the measurement and its interpretation can be tested using two further corpora as well as an experimental study. We conclude by discussing future developments in the field.

**Supplementary Information:**

The online version contains supplementary material available at 10.3758/s13428-020-01511-3.

Numerous group and category memberships shape our everyday interactions (Tajfel & Turner, 1979; Turner, Hogg, Oakes, Reicher, & Wetherell, 1987). In many everyday situations, our actions and interactions are guided by the norms of the group membership that is psychologically salient within a specific social context (Hogg & Reid, 2006). As a result, the salient social category affects our attitudes (Reynolds, Turner, Haslam, & Ryan, 2001), cognitions (Haslam, Oakes, Reynolds, & Turner, 1999), emotions (Doosje, Branscombe, Spears, & Manstead, 1998), and behavior (Shih, Pittinsky, & Ambady, 1999). Hence, understanding which social group membership is salient within a social context provides valuable insights into the socio-cognitive underpinnings of social behavior and allows researchers to explain differences between situations and among different individuals. However, we are currently lacking methods to determine which social group membership is salient in a given situation, thereby mostly limiting research to experimental studies. By their very nature, experiments can neither provide insights into the dynamic aspects of group membership, nor elucidate their effects in naturalistic settings.

We argue here that advances in computational approaches and natural language processing allow us to create standardized methods that can be relatively easily constructed. Through the use of linguistic data, they can be employed in a wide range of settings, potentially bridging experimental, qualitative, and big data computational approaches. By opening up the field to naturally occurring data such as social media posts, the method also provides an opportunity to study social phenomena “in the wild” and at scale (e.g., Callon & Rabeharisoa, 2003), and create applications for the common good.

## Context dependence in social group memberships

Given the importance of social groups in many different spheres of life, social categorization effects have been examined in a wide variety of disciplines within psychology, including developmental psychology, clinical psychology, and organizational psychology (Haslam, 2014), as well as in fields outside of psychology, such as economics, education, political science, and sociolinguistics (Reicher, Spears, & Haslam, 2010). Much of this research has been conducted within the social identity tradition (Hornsey, 2008), examining the effects of social identification, social identity salience, group prototypicality, and related constructs on attitudes, cognitions, emotions, and behavior.

In this research tradition, the various group and category memberships that we hold are understood to form an important part of our self—our social identity. This conceptualization of the self recognizes that groups and their norms affect our cognitions, emotions, and behavior in many social situations in a way that cannot be explained through the personal, idiosyncratic aspects of our self, our personal identity. In particular, social identity can help us to understand collective behavior (e.g., protests), collective emotions (e.g., national pride), and shared attitudes (e.g., prejudice).

An important aspect of social identity is the notion of context dependence. Rather than exerting a constant influence on individuals, social identities are sensitive to changes in the social context. In particular, self-categorization theory (SCT; Turner et al., 1987) proposes that the social context makes a particular group membership salient, thereby activating the associated norms and values. As a result, the salient identity guides the behavior, cognitions, attitudes, and emotions of group members, particularly of those members that identify highly with the group.

It is important to note here that the construct of social identity salience refers to the context-dependent cognitive access to a particular in-group identity and the internalized norms and values associated with this group (Turner, 1981). This is in contrast to the cognitive-linguistic construct of word (or category) salience that refers to the cognitive activation of a word and a network of related words and concepts (Schmid & Günther, 2016). Although both constructs share the basic idea of cognitive activation, they differ in the self-relevance of the unit. For example, the word/category of “child” can be made salient in any person, including non-parents. In contrast, a parent identity can only become salient in those who self-categorize as a parent and who have begun to internalize the norms and values associated with this identity.

Importantly, the group norms and values that are accessed when the identity becomes salient are based on the comparative context in which group members find themselves. In particular, group norms and values that differentiate the salient social identity from other relevant groups are highlighted within that comparative context (Turner, Oakes, Haslam, & McGarty, 1994). For instance, Haslam, Oakes, Turner, and McGarty (1995) found that Australians emphasized traits such as being sportsmanlike, and de-emphasized traits such as happy-go-lucky, when describing the in-group in a comparative context with Americans. Based on evidence from studies on self-stereotyping and group polarization, Turner et al. (1994) concluded that self-categorization is “comparative, inherently variable, fluid, and context dependent” (p. 458). Consequently, identity salience—and the norms that are activated—do not operate in an absolute sense in a social vacuum. Rather, social identities become salient in a comparative context, with intergroup-differentiating norms guiding in-group members’ thoughts, emotions, and behavior.

Research on a multitude of social phenomena has found support for the wide-reaching effects of social identity salience, including on helping behavior (Levine et al., 2005), cooperation (Kramer & Brewer, 1984), voting behavior (Bryan, Walton, Rogers, & Dweck, 2011), crowd behavior (Alnabulsi & Drury, 2014), performance (Afridi, Li, & Ren, 2015; Shih et al., 1999), organizational innovation (Mitchell & Boyle, 2015), sexism (Wang & Dovidio, 2017), olfactory judgments (Coppin, Pool, Delplanque, Oud, Margot, Sander, & van Bavel, 2016), and selective forgetting (Coman & Hirst, 2015), among numerous others. Although not always explicitly acknowledged, many of these studies exploit a particular comparative context to emphasize specific aspects of an identity. For example, Levine et al. (2005) deliberately placed football fans in a comparative context with hooligans, thereby highlighting the prosocial side and sportsman-like conduct of football fans, who subsequently were more likely to help a fellow football fan in need. Similarly, Coppin et al. (2016) found that Swiss people reported a more intense odor of chocolate than non-Swiss participants when primed with the Swiss identity, presumably because Switzerland is famous for its high-quality chocolates compared with most other nations. However, a comparison with Belgian participants who may be similarly proud of their country’s chocolates may have yielded different results.

In addition to experimental studies showing the power of social identity salience, a growing research area is the use of social identity principles to advance mental health and well-being, offering the potential of a “social cure” (Jetten, Haslam, & Haslam, 2012; Haslam, Jetten, Cruwys, Dingle, & Haslam, 2018). Research in this tradition shows that multiple group memberships generally have a positive effect on mental health (Haslam, Cruwys, Haslam, Dingle, & Chang, 2016), and make individuals more resilient in times of change, such as following a life-changing illness (Haslam, Holme, Haslam, Iyer, Jetten, & Williams, 2008) or the birth of a child (Seymour-Smith, Cruwys, Haslam, & Brodribb, 2017). Although research initially focused on the number of self-reported groups and the level of identification with such groups, more recent models and studies have started to look at the salience of group memberships (Cruwys, South, Greenaway, & Haslam, 2015) and the interplay between different social identities (Haslam et al., 2016), as well as the acquisition and loss of identities over time (Best et al., 2016; Frings & Albery, 2015).

Similarly, organizational psychology has long been interested in understanding the interplay of multiple organizational identities held by individual employees on performance, cooperation/conflict, and well-being (Haslam, 2004; Steffens, Haslam, Schuh, Jetten, & van Dick, 2017; Wegge & Haslam, 2014), as well as in the acquisition and loss of identities over time, such as in the case of organizational mergers (van Leeuwen & van Knippenberg, 2014) or the retirement of employees (Lam et al., 2018). Organizational and work group identification/commitment are central variables in this line of research, and are known to be affected by salience in a given situation (Van Dick, Wagner, Stellmacher, & Christ, 2011).

With social identity research advancing in applied areas, the dynamic assessment of a salient identity in natural contexts becomes more pressing. Important questions remain regarding the interplay between different identities over time, the factors that enhance or undermine salience in natural contexts, and the integration of different social identities into the self-concept. In particular, longitudinal data assessing the relative salience of potentially competing identities is lacking, as the measurement of social identity salience is largely confined to the laboratory.

## Current assessments of social identity salience

The majority of studies considering the salience of an identity are of an experimental nature where salience is manipulated or measured indirectly. Although experimental studies have undoubtedly provided important insights into the effects of social identities, they are not well suited to study the impact of social identities in naturalistic settings, or the dynamic interplay of different identities over a longer period of time. However, the emphasis on experimental studies is unsurprising given the difficulties in assessing salience through self-report or observation.

### Self-report measures

Although some researchers have attempted to measure salience with survey items or as part of qualitative studies (e.g., Haslam et al., 1999; Lobel & St. Clair, 1992; Neville & Reicher, 2011; Yip, 2005; see also Abdelal, Herrera, Johnston, & McDermott, 2009), two main difficulties arise: measurement reactivity and lack of introspection.

Items or interview questions that aim to assess the salience of an identity of interest to the researcher may induce measurement reactivity in the participant (see Brenner & DeLamater, 2016, for reactivity in the self-reporting of identity-related behavior); that is, they may unintentionally make an identity salient, leading to over-reporting. For instance, asking a participant whether they are, at the moment, thinking of themselves as a student is likely to make the very identity salient that the question intends to assess.

Alternatively, an open question may be asked where no particular identity is mentioned and the participant is free to list the identity that is salient at that very moment. The difficulty here is that participants may struggle to provide an answer. Salience is thought to be largely an outcome of an automatic (“fluid”) process of self-categorization (Turner, Oakes, Haslam, & McGarty, 1994), and participants may lack the introspection to answer the question (see Silvia & Gendolla, 2001).

Another commonly chosen route is to assess social identity salience with social identification items (e.g., Callero, 1985; Phalet, Baysu, & Verkuyten, 2010; Reicher, Templeton, Neville, Ferrari, & Drury, 2016), despite clear theoretical differences between the two constructs (McGarty, 2001). In addition to these methodological difficulties, self-report measures are also not well suited to study the dynamics of social identities within a naturalistic setting, or over longer periods of time.

### Observational inference

An alternative approach to self-reporting is the observational inference of the identity that is most likely to be salient in a given moment. This approach is based on the idea that social norms that are activated by the salient social identity are guiding the behavior of group members, thereby creating homogeneity in in-group behavior and differentiation from out-group behavior. For instance, observing a crowd of football fans cheer on their team, or a group of protesters march towards parliament, may lead to the inference that the social identity of football fan or political activist, respectively, is salient.

This approach has the advantage that situationally induced changes in salience can be studied in a dynamic real-world context. Drury and Reicher (1999), for example, used video footage of intergroup dynamics, and observed that the actions of authorities created a shift in salience from small groups (“cliques”) towards a more united group of “protesters” (see also Reicher, 1996). Using homogeneity in behavior as an indicator of identity salience has provided powerful insights into the dynamics of identity salience in natural settings, with important implications for applied areas such as the policing of crowds (e.g., Stott, Adang, Livingstone, & Schreiber, 2006). However, this method has so far only been used in observational studies that analyze groups as a whole rather than individual members, and can therefore not answer questions on individual-level dynamics in social identity salience. The behavior also needs to be prominent enough to be recognized as originating from a particular social identity. Hence, the behavior studied is of a nature that does not lend itself to be used in standardized methods that go beyond an idiosyncratic situation.

### Indirect measures of salience

The idea that social identity salience produces measurable effects from which the strength of social categorization can be inferred has also been used in laboratory paradigms. The most prominent of these measures is the “Who Said What?” paradigm (WSW; Taylor, Fiske, Etcoff, & Ruderman, 1978). This paradigm uses a memory task where “speakers” that have different attributes (e.g., skin color) related to a social category of interest are presented making a number of different statements. After the presentation of the speaker–statement pairs (“discussion phase”), participants are asked in an “assignment phase” to recall which speaker made which statement. Salience of the social category is inferred from an error-difference measure that compares the number of within- and between-category errors. The more within-category versus between-category errors that occur, the stronger the salience of the social category. Klauer and Wegener (1998) modified the initial paradigm and introduced a multinomial processing tree to account for different cognitive processes that might affect the error-difference measure, thereby increasing its power and validity. The paradigm is commonly used in controlled laboratory experiments, but is now increasingly employed in online experiments too (e.g., Flade, Klar, & Imhoff, 2019).

More recently, event-related potentials (ERPs) have been used to detect a neural categorization effect that responds to changes in contextual social identity salience (Domen, Derks, van Veelen, & Scheepers, 2020). However, neither the WSW paradigm nor ERPs can be used to study the dynamic aspects of social identity salience in real-world contexts.

### Computational linguistics

Outside the social identity tradition, computational linguistics approaches have started to assess whether an individual is part of a particular social group, such as being a man or woman (Newman, Groom, Handelman, & Pennebaker, 2008; Schwartz et al., 2013), Republican or Democrat (Sylwester & Purver, 2015), or Christian or Atheist (Ritter, Preston, & Hernandez, 2014). For instance, the Isis toolkit uses a combination of natural language processing and authorship attribution to predict age categories (e.g., child/adult) and gender categories (male/female) with remarkably high accuracy (80%) using short texts (e.g., from chat rooms; Rashid et al., 2013). These studies have taken advantage of the availability of large corpora of text, such as social media posts. By combining natural language processing techniques and machine learning approaches, they have created classifiers that distinguish between the groups of interest based on the message that an individual wrote (see Nguyen, Doğruöz, Rosé, & de Jong, 2016, for a review).

There are two problematic aspects with most computational linguistics studies of this kind for assessing a salient social identity. Firstly, the training of a classification model on two mutually exclusive groups using naturally occurring data invites several confounds. Differences in language use between the studied groups may be due to differences in group members’ demographics or personality that impact language, such as education, social class, age, assertiveness, conscientiousness, and so on (Pennebaker & King, 1999; Wolfram & Schilling-Estes, 2005). Language differences may also be due to differences in the topics that the groups discuss rather than group membership per se (Rickford & McNair-Knox, 1994).

Secondly, the models do not take into account the dynamic nature of social identity salience. Instead, they implicitly assume that groups exert their influence constantly. For instance, models that are trained to detect gender in language are assumed to be valid in all situations, whether gender is salient in that context or not. These models are therefore not well positioned (and neither were they intended) to assess the salience of a social identity, and dynamic changes between different social identities.

Although current computational models do not—to the best of our knowledge—assess the salience of social identities, they open up the possibility of using natural language processing techniques and machine learning to assess social identity salience in naturally occurring text data.

## Automated Social Identity Assessment (ASIA)

Assessing the salience of a social identity in a dynamic, theory-driven way would allow researchers to study how social identities operate in complex and changing environments where several identities may compete. Ideally, the assessment method should be relatively easy to use, be specific to the social identities of interest to the research, and allow for comparisons across contexts.

Given the large number of different social identities, a single tool is unlikely to allow for a valid assessment of each of them. However, it may be possible to create specific models based on the theoretical assumption that all social identities affect behavior through their norms and values once they are salient. Individuals are active users and communicators of their social identities (Klein, Spears, & Reicher, 2007). They strive to communicate a desired social identity to others by behaving in line with group norms: both towards the in-group to assert their group membership, and towards out-group members to achieve intergroup differentiation (Tamburrini, Cinnirella, Jansen, & Bryden, 2015). The in-group homogeneity and intergroup differentiation created by the process of self-categorization can potentially be exploited in a binary classification model to assess which of two identities is salient.

Based on the successful use of linguistic information for group classification as demonstrated by computational sociolinguistic approaches (Nguyen et al., 2016), and the wide availability of written text data for research (e.g., from online forums/social media, emails, diaries, official documents, historical texts), we focus on linguistic style as the behavioral indicator of identity.

Sociolinguistic theories have long held that each of us is part of a large number of different social groups and categories that influence our language use, in terms of both vocabulary and style (e.g., Coupland, 2007; Le Page, Le Page, & Tabouret-Keller, 1985). Early theorists in sociolinguistics such as Labov (1968/2006) suggested a social dimension to intra-individual language use. In particular, he suggested that social variables would affect *stylistic* choices. Such intra-individual style shifts can be observed, for instance, in code switching, the “alternations of linguistic varieties within the same conversation” (Myers-Scotton, 1993, p. 1). This can take many forms, from switching from one language to another within the same sentence, to moving from a formal to an informal style during a conversation. Here we propose a computational model that exploits such shifts, or switches, that are driven by self-categorization in order to assess which identity is salient in a given moment.

### Building an ASIA tool

In the following, we describe an analytical protocol for training and validating an ASIA tool. These steps include guidance on ethical considerations for the selection of training and testing material as well as steps to establish the quality of the measurement. We consider both aspects—ethics and validation—to be central to the analytic protocol, and the measure more widely.

1. Ethical considerations
2. Selection of the training dataset
3. Quantifying stylistic features from text
4. Training the model
5. Cross-validating the model
6. Generalizability across platforms
7. Construct validity
8. Concurrent validity

We will explain each step in general and then provide an example with a proof-of-concept case: parent versus feminist identity salience. For our proof-of-concept case, we chose two large-scale social groups that show a good overlap in membership but distinctiveness in their prototype as well as a good online presence. This allows us to test for between-group differences as well as within-person shifts in linguistic style. Furthermore, both identities play an important role in the lives of a large number of people. In fact, a parent identity may be the single most widely shared social identity in the world, with about 75–80% of men and women over the age of 40 having at least one biological child (OECD, 2018; Monte & Knop, 2019) and others becoming parents, for instance through adoption or shared living arrangements. Being a parent affects many parts of a person’s life including work–life balance, economic decision-making, and health and well-being. There is also currently a strong research interest in feminist identities, partly due to the #MeToo movement as well as debates around transgender rights in relation to women’s rights.

A hands-on Jupyter Notebook tutorial with annotated code for the proof-of-concept case can be found on GitHub: https://github.com/Identity-lab/Tutorial-on-salient-social-Identity-detection-model

### Step 1: Ethical considerations

Assessing salient identities in naturally occurring text data raises two main ethical concerns: (i) Is it ethical to assess the specific social identities in question? (ii) Can data from online sources be ethically used to train, test, and validate the model?

Individuals may choose to hide their social identities for legitimate reasons. For instance, revealing a stigmatized social identity may place individuals in physical danger and may expose the individual to discrimination and ostracism (Quinn, 2017). Furthermore, assessing salient social identities indirectly—potentially without awareness or consent from the individual—may undermine an individual’s privacy rights and make them vulnerable to financial and social discrimination (Bodie, Cherry, McCormick, & Tang, 2017). Hence, a tool which can identify salient identities is susceptible to misuse. We therefore impart on researchers and practitioners a responsibility to consider the specific domains in which they employ ASIA as a tool. In particular, foreseeable harm to individuals needs to be considered before research commences and, in line with APA and BPS ethical guidelines, steps need to be taken to minimize any risk of harm (American Psychological Association, 2017; British Psychological Society, 2018).

Questions of privacy and harm also pertain directly to the selection of training and testing datasets. Based on APA guidelines, online material such as online forum posts should only be used where either explicit consent from the user has been given or where the material can be reasonably considered to be in the public domain. Social media users sometimes find it difficult to apply appropriate privacy settings to their accounts and are often unaware of a platform’s terms and conditions (e.g., Facebook; Liu, Gummadi, Krishnamurthy, & Mislove, 2011). Hence, researchers cannot simply assume that the user intended the information to be in the public domain. It is therefore advisable to focus on public online forums rather than social media platforms. Public online forums also have the advantage that users are usually anonymous. In contrast to Facebook, Twitter, Instagram, and similar social media platforms, users in public online forums are rarely using their actual name but are instead encouraged to use an alias (a user ID) with little personally identifying information in the form of metadata (e.g., demographics, geo-location) available on users. Where a platform’s terms and conditions do not explicitly state that third parties can use posts (e.g., for research), it is advisable to contact the platform owners to ask for permission to avoid copyright and privacy infringements. Publications and data that are made available online should exclude original posts and user IDs. Text from original posts can be easily traced back to a user via search engines—and may unintentionally harm the user, particularly where a quantitative assessment of the salient identity is linked to the text data in the dataset (for a wider discussion of the use of social media data from a user perspective, see Beninger, 2017).

#### Proof of concept: Ethics

For our proof-of-concept case, in which we aim to detect parent and feminist identity salience, we chose identities that are widely held and not highly stigmatized. We collected the datasets with permission of the platform owners (Mumsnet UK, Netmums UK) or where permission for research use is granted by the terms and conditions (Reddit). All three platforms explicitly inform users that any content created is in the public domain and rights are owned by the platform rather than the user. Furthermore, forums on all these platforms are clearly signposted as being in the public domain rather than a place for private conversations, and therefore do not fall under the principle of “reasonable expectation of privacy”. For instance, Netmums UK calls their forum “Coffeehouse” to indicate its public nature. All of these platforms allow private messaging between users, thereby highlighting the distinction between public and private channels. No private messages are included in any of our datasets. All five studies presented here received ethical approval from the University of Exeter psychology ethics committee.

### Step 2: Selection of training dataset

Training a good classification model depends heavily on the quality of the data. Training data may introduce biases due to the particular demographic of users on a chosen platform and within sub-forums (Nguyen et al., 2016). It is therefore advisable to either train on data from a platform that is relatively diverse, or alternatively, to validate the trained model on platforms that are known to differ demographically from the original platform (see Step 6) to ensure that findings are not due to the biased nature of the training data.

In order to train an ASIA tool, posts from two intersecting—rather than mutually exclusive—groups need to be identified. Intersecting groups are those where a person can, in principle, be a member of both groups. This includes group memberships that may, on occasion, be in conflict (e.g., parent and work identities), and those where one group membership is part of a superordinate identity (e.g., Asian American). In contrast, mutually exclusive group memberships are those where a simultaneous membership in both groups would be considered a serious violation of group norms (e.g., vegetarian and “meat-eater”, Republican and Democrat, Christian and Atheist).

For many larger groups, specific platforms exist (e.g., Mumsnet and Netmums for parents in the UK). These platforms may also host sub-forums for related social groups. For instance, Mumsnet UK hosts one of the largest feminist forums in the UK as one of their sub-forums. Some platforms, such as Reddit, provide forums for a wealth of social groups. This has the advantage that it is relatively easy to identify users with more than one social identity of interest—and allows for within-participant testing that controls for demographics and stable traits (see Step 5). However, care needs to be taken to ensure that the forum is likely to consist of individuals holding the group membership of interest rather than a combination of different groups debating a shared topic of interest. Often, forums include a number of non-members (e.g., moderators, trolls, and bots). This should not pose a problem as long as the vast majority consists of group members, and data cleaning procedures are undertaken to reduce the impact of non-member messages.

#### Proof of concept: Study 1 data

The online forum data for training our model were gathered from the online website Mumsnet UK (www.mumsnet.com/talk), the largest parent online network in the UK, with the kind permission of Mumsnet UK. This site provides different sub-forums in which users can discuss particular topics and themes. We analyzed posts from two sub-forums, “Being a Parent” and “Feminism”. The posts were collected in September 2012 from 2500 threads per sub-forum. Every person who wishes to contribute to Mumsnet UK is required to create a user account with a unique user ID. Hence, posts from the same author can be matched by the user ID, irrespective of the sub-forum in which they were posted.

Overall, our sample consists of *N* = 620,866 posts written by *N* = 19,745 different users*.* A total of *n* = 394,205 posts from *n* = 12,688 users were collected from the “Being a Parent” sub-forum and *n* = 226,661 posts from *n* = 9940 users from the “Feminism” sub-forum, with *n* = 2883 of these users having posted in both forums. Although it is not possible to extract demographic data, Pedersen and Smithson (2013) found in their study of Mumsnet users that the majority were mothers (97%), between 31 and 40 years of age (61%), with a high level of education (34% having a university degree). Since this is not a representative sample of mothers/parents or feminists, we chose platforms and users with different demographics from Mumsnet for our validation studies. To reduce influences from non-member messages, we excluded posts that only included an administrative message from Mumsnet rather than a genuine message by the user (e.g., “Message withdrawn” or “Message deleted by Mumsnet”), or messages that did not include words (e.g., only an emoji or picture). No messages from bots that identified themselves as a bot were found.

### Step 3: Quantifying stylistic features from text

For relatively easy feature extraction from texts, the software Linguistic Inquiry and Word Count (LIWC; Pennebaker, Booth, & Francis, 2007) can be used to quantify linguistic features. LIWC is a widely used expert-based system that maps each word to one or more linguistic features so that documents are represented as a normalized frequency of each feature. LIWC mappings have been developed and refined over a number of years by panels of researchers in psychology and language, and are based on a variety of corpora (Pennebaker, Chung, Ireland, Gonzales, & Booth, 2007; Tausczik & Pennebaker, 2010). Although closed-vocabulary approaches such as LIWC that provide simple category counts are sometimes considered less predictive than open-vocabulary approaches (Schwartz et al., 2013), LIWC has the advantage that it is a widely available software and can be used for short documents. Hence, results can be easily replicated and ASIA tools can be created by those who are new to natural language processing techniques.

LIWC provides counts for “part-of-speech categories” and “topical categories” (Schwartz et al., 2013). Part-of-speech categories represent words used generally across multiple contexts such as different grammatical categories (e.g., different types of pronouns) and basic psychological categories (e.g., time words, positive/negative emotion words). We use these categories as *style* features in our studies, that is, features that reflect *how* a message is written rather than which topic is discussed. Here, it needs to be noted that LIWC has a number of hierarchical features (e.g., the negative emotion category includes the sub-categories anxiety, anger, and sadness). To avoid redundancies, it is advisable to exclude either the higher-order category or all lower-order categories. The choice of level will, for instance, depend on the frequency distributions for each feature. Where low frequencies occur in the lower-order categories, a higher-order category may lead to more reliable results.

In line with sociolinguistic theory, we do *not* include topical categories (e.g., family, work, money) but focus on stylistic variation. By excluding topical categories, the initial accuracy is likely to be lower than when including them. However, the risk with topical categories is that particular words (e.g., “child” for a parent identity or “women” for a feminist identity) will dominate the classification model. Hence, excluding topical categories reduces the risk of overfitting and increases the chance that the salient identity can be detected irrespective of topic, in a variety of settings. The use of “bag of words” indicators, such as LIWC features, rather than individual words also contributes to the robustness of the model.

#### Proof of concept: Study 1 features

We extracted 44 different non-redundant style features from each text. These include words per sentence (WPS), grammatical features (function words, various pronouns, articles, prepositions, verbs and so on, tenses, quantifiers, numbers), basic psychological categories (e.g., time words, long words of six characters or more, positive emotions, negative emotions, swear words, negation, assent, insight, causality, discrepancy, tentative, inclusive words), and punctuation (e.g., semicolon, apostrophe).

### Step 4: Training the model

A range of machine learning approaches is available for a supervised learning task where data need to be classified into two known groups, such as logistic regression, support vector machines (SVM), decision tree-based classifiers, and neural networks. In contrast to some of the other machine learning approaches, logistic regression relies on linear relationships between predictor variables and the outcome. A key advantage of logistic regression is that it results in a clear regression equation where coefficients can be interpreted with regard to both their weight (“importance”) for the classification and the direction of the effect. As with other regression approaches, coefficients need to be interpreted as a pattern rather than individually.

As part of model training, all stylistic features are included within the model. The fitting procedure is then allowed to ignore non-informative features, thereby identifying those features that are most predictive of differences between forums. The overall performance of the model can be estimated through the area under the ROC curve (AUC), the recommended way to report prediction accuracy for dichotomous variables (e.g., Kosinski, Wang, Lakkaraju, & Leskovec, 2016). This provides a measure of how well the model separates between the two classes, with AUC = .50 equivalent to guessing (i.e., no class separation) and 1 as perfect separation. To estimate standard errors, bootstrapping can be used if the dataset is relatively large.

To achieve reliable classification, we recommend where possible that very short posts be excluded from the dataset. Very short posts are unlikely to be informative enough to allow a correct classification. However, excluding these posts—particularly if a large number of posts are very short—reduces generalizability to other datasets and interferes with robust evaluation. An empirical approach to get a sense of which cutoff for post length is useful is to estimate a model for each cutoff point (i.e., all messages, messages with two or more words, three or more words, and so on), then drawing a graph with the cutoff point as the *x*-axis and associated AUC as the *y*-axis. In combination with a histogram of the word count, this graph helps the researcher to find a trade-off between accuracy and generalizability that is in keeping with their research aim.

#### Proof of concept: Study 1 method and results

**Fig. 1:**
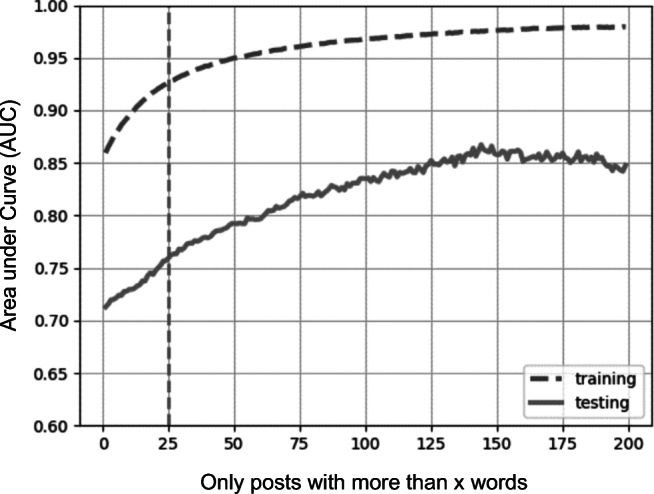
Predictive performance (AUC) by word count cutoff for Study 1 (Mumsnet data); the hyphenated vertical line indicates Quartile 1

For our proof-of-concept case, we estimated a model for each cutoff point (see Fig. 1). Our lower quartile for word count is Q1 = 25 words. Figure 1 shows that using posts with 25 words or more would give us an AUC > .90 for our training and AUC > .75 for testing. We therefore decided to restrict ourselves to the most informative 75% of posts by removing the first quartile of posts.

After removing posts of 24 or fewer words, our training sample consists of *N* = 461,371 posts written by *N* = 18,031 different users*,* of which *n* = 306,924 posts stem from *n* = 11,780 users in the “Being a Parent” sub-forum, and *n* = 154,447 posts stem from *n* = 8584 users in the “Feminism” sub-forum, with *n *= 2333 of these users having posted in both forums.

To train our model, we used a bootstrapping procedure: We randomly sampled 20 subsets of 100,000 posts from the complete training dataset. For each subset, half of the posts were randomly sampled from the full set of “Being a Parent” posts and the other half were randomly sampled from the full set of “Feminism” posts. Posts from users who had only posted in one of the two forums, along with posts from those who had used both forums, were included in the training dataset. This “between-forums” design is used to enable the widest possible sample of parents and feminists on the platform to be included in the training of the model.

**Fig. 2:**
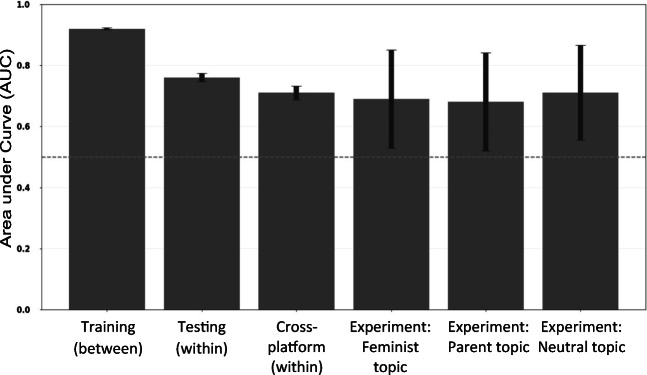
Predictive performance (mean AUC) for training and test data (Mumsnet), cross-platform test (Reddit), and experimental data; error bars show 95% confidence intervals, the dotted horizontal line indicates no class separation (i.e., “guessing accuracy”)

A logistic regression model with all 44 style variables as predictors and identity (parent vs. feminist) as outcome, using posts of 25 words or more, yields a very good prediction accuracy of mean AUC = .92, *SE* = 0.002 (see Fig. 2 for AUCs and 95% confidence intervals for all training and test models). These results show that the pattern of stylistic features of the two identities is sufficiently distinct that it is possible to accurately classify from which group a text stems.

**Fig. 3:**
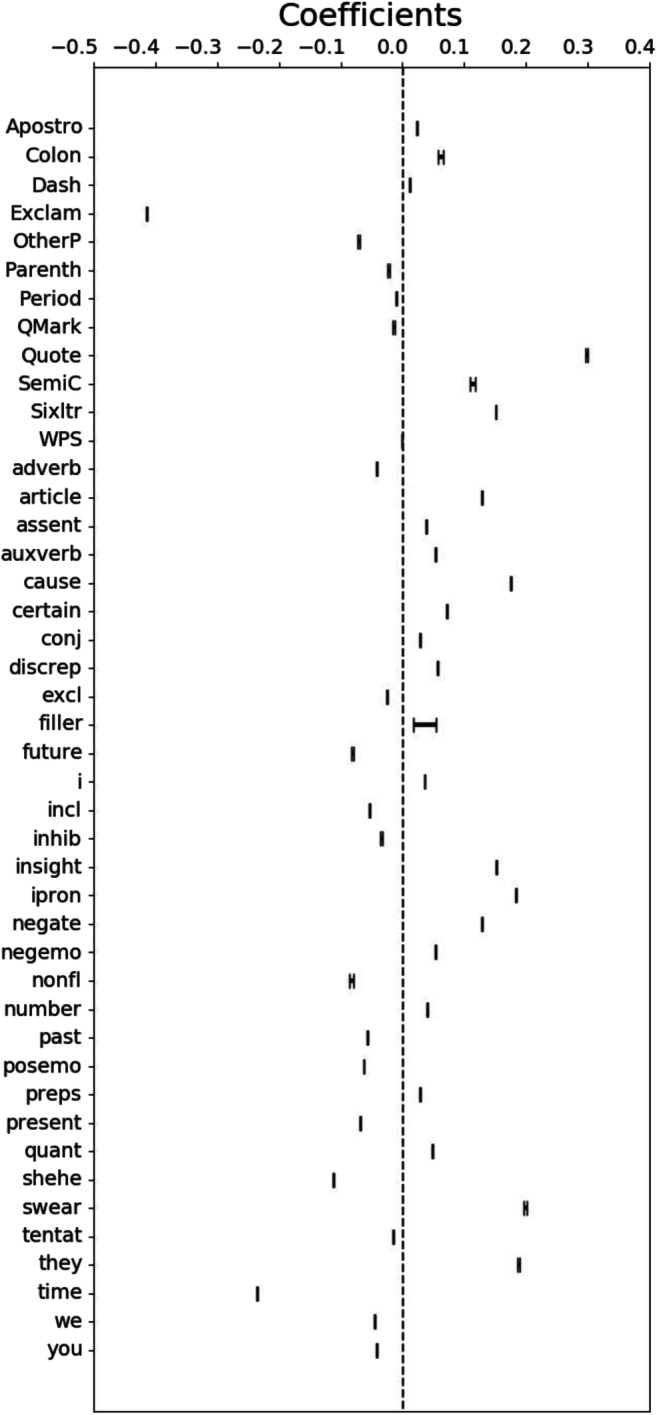
Standardized coefficients with standard error for Study 1 training data (Mumsnet); negative coefficients (on the left) indicate parent identity salience, positive coefficients (on the right) indicate feminist identity salience

Figure 3 provides the coefficients for each linguistic indicator and their standard errors. The overall pattern suggests that a feminist identity (positive coefficients) is expressed through a more intellectual style (e.g., use of long words (sixltrs), articles, semicolons, words related to causality and insights) with more negative connotations (e.g., negating words, negative emotions, swear words) than the parent identity. In contrast, the parent identity is characterized by a more informal style (e.g., use of exclamation marks, non-fluency), with a focus on specific individuals (he/she) and events (time words) and the expression of positivity and inclusiveness (posemo, incl). It needs to be noted here that some indicators (e.g., swear words) can be highly predictive of one category over another when seen in conjunction with the other indicators—however, they are a relatively rare occurrence overall (see Supplementary Material for word frequencies of the five strongest indicators for each identity). Hence, using the whole pattern rather than individual words provides a more robust measure of social identity salience.

### Step 5: Cross-validating the model on within-participant data

A straightforward way to test whether the classification is driven by confounds such as demographics (e.g., social class, level of education) or personality differences between members of the two social groups is to use a within-participant design. A sub-sample is used that consists of one randomly drawn post from each of the two forums, written by users who have posted in *both* forums. A random post per user and forum is used rather than all posts in order to avoid bias; that is, users who differ in personality or demographics (e.g., education) may post more in one forum than another. Using one post per forum for each user means that we can keep such differences between users constant, analogous to a within-participant design.

Hence, a successful classification of posts to forums cannot be explained by demographic factors or other stable traits, since posts from each forum were written by the same set of users. This test is also interesting from a theoretical perspective, as it allows us to examine whether systematic *intra-individual* style shifts occur when the social context changes, in line with changes in social identity salience.

#### Proof of concept: Study 2 – Validating on within-user data

In the within-participant test stage, the trained classifier was cross-validated 20 times on posts from *n* = 2333 users who had posted in both the feminist and parent forums, using one random post from each of the two forums for each user for each round of cross-validation.

Testing the trained model from Study 1 on the within-participant data produces a good prediction accuracy of mean AUC = .76, *SE =* 0.01. This result shows that posts by the same user can be accurately classified, indicating intra-individual style shifts in line with changes in social identity salience. Importantly, the test sample of individuals who had posted in both forums controls for stable individual differences between the two social groups such as age, education, social class, and personality differences.

### Step 6: Testing generalizability across online platforms

Platform effects are a common problem for computational models (Pearce et al., 2020): a model trained on data from one source (e.g., Twitter) may not be accurate in classifying data from other sources (e.g., Reddit), thereby undermining generalizability. Such platform effects may be due to differences in restrictions placed on posts (e.g., word count, availability of emojis), moderation rules (e.g., no swearing), and other factors (e.g., location in the UK or USA). Generalizability may also be undermined by a lack of representativeness of the users for the groups as a whole (Nguyen et al., 2016). Some demographics are overrepresented online, and this is additionally compounded by a self-selection towards particular platforms. Demographic data about individual users is rarely available. It is therefore important to test for generalizability across platforms by testing the trained model on one or more datasets of the same two groups from different platforms wherever possible, ideally on platforms with a different demographic user profile (where aggregated user information is available).

#### Proof of concept: Study 3 – Generalization across platforms

In order to test whether a model trained on parent and feminist forums on Mumsnet UK, with its particular demographic, generalizes to a different platform, we collected data from a parent and a feminist forum on Reddit. Reddit is an American platform with 50% of visitors from the USA, 8% from the UK, and 8% from Canada, as well as various other countries (Clement, 2019). A survey by Barthel, Stocking, Holcomb, and Mitchell (2016) suggests that 64% of American Reddit users are aged 18–29, 29% are 30–49 years old, and 7% over 50 years old. American Reddit users are White non-Hispanic (70%), Hispanic (12%), Black non-Hispanic (7%), or other non-Hispanic (11%). The majority of American Reddit users have a college degree (42%) or some form of college education (40%). No demographic data for the two subreddits of interest are available to the best of our knowledge.

**Fig. 4:**
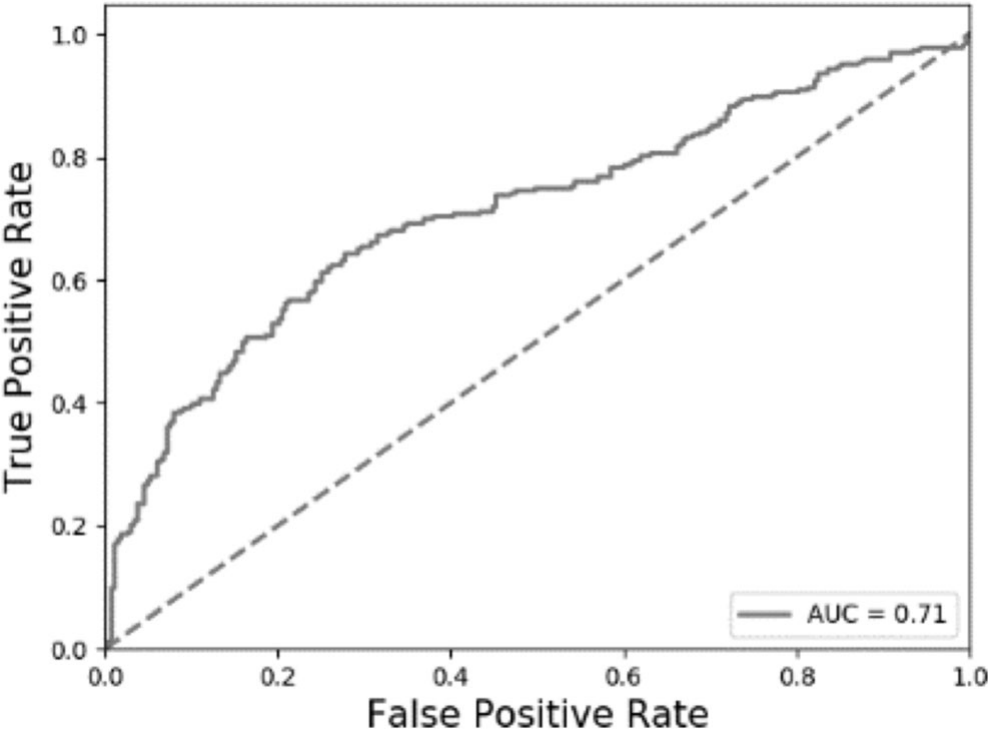
ROC for cross-platform testing (Study 3)

Data were collected from r/parenting and r/feminism using posts written between January and December 2018. Moderator messages and messages from bots who self-identified as such were cleaned from the data (see detailed tutorial for information), and only posts of 25 words or more were included in this dataset. To validate our model across platforms, we again used a within-participant design where only users that had posted at least once in both r/parenting and r/feminism were included. The dataset includes 49,640 posts written by *n* = 263 users. We randomly drew one post per forum per user for each of the 20 cross-validation models. We find that the model still performs well, with a mean AUC = .71, *SE* = 0.01 (see also Figs. 2 and 4; a confusion matrix is provided in Supplementary Materials).

Our finding shows that performance is only slightly lower across platforms, even when demographics and other stable characteristics are controlled for. This result indicates that linguistic style is not simply conformity to a local style of a particular online community, for instance as a result of accommodation or other local social influence mechanisms (Danescu-Niculescu-Mizil, Gamon, & Dumais, 2011; Giles, Taylor, & Bourhis, 1973), but a wider expression of a salient identity that is shared amongst people from different demographics, and even different countries.

### Step 7: Construct validity

So far, the steps have tested whether posts from two forums that are related to social identities (1) differ sufficiently in linguistic style that a good classification can be achieved, (2) differ in a group prototypical way even when written by the same person, and (3) reflect a prototypical writing style that goes beyond local norms/accommodation.

Forum posts, however, cannot fully test construct validity, that is, whether it is really social identity salience that causes the shift in linguistic style. More specifically, an analysis of naturally occurring data is open to confounding variables such as differences in topics and audiences between the forums that may explain the differences in style.

An experimental study that manipulates social identity salience while controlling for audience, topic, and other confounds is needed to ensure that it is, indeed, social identity salience that is being assessed. Salience can be relatively easily manipulated experimentally (see Haslam, 2004, for a discussion of several methods), and the ability of the classification model to assess the salience of the identities in question can therefore be experimentally tested.

#### Proof of concept: Study 4 – Experimental validation

In Study 4, we use the classifiers trained in Study 1 (Mumsnet data) on a new dataset from an online experiment. The experiment allows us to use self-reported social identities as the criterion, rather than the proxy “forum”. Importantly, by focusing only on those who self-report both identities, salience of identity can be manipulated in order to test whether our model can, indeed, predict which social identity was salient during writing. The experiment also allows us to control for conversational topic, exclude variation in audience as the source of differences in style, and control for demographic and other individual differences. We recruited participants from websites other than Mumsnet to test whether the classifier trained on Mumsnet generalizes to other contexts and demographics.

***Participants and design.*** We calculated the target sample size (total *N* = 42; Goksuluk, Kormaz, Zararsiz, & Karaagaoglu, 2016) for testing our classifier from Study 1 with power of .80 and an AUC of .71 (see Step 6–cross-platform, within-participant test), assuming an equal ratio of parents and feminists. Participants were recruited via advertising in forums such as Netmums UK (an alternative platform to Mumsnet), Reddit (r/feminism), Facebook, and Twitter, and through a paid online recruitment platform in the UK, Prolific Academic—the latter to include participants who are not active in online forums. Notably, the study was not advertised on Mumsnet, and only *n* = 4 participants (9 %) indicated that they had used Mumsnet.

A total of *N =* 43 native English speakers who indicated both a parent and feminist identity participated in the online study. The vast majority of participants were female (*n* = 41; 95%). Participants were between 26 and 69 years old (*M* = 42.05, *SD* = 10.61) and had between one and four children (*M* = 2.07, *SD* = 0.86). The majority of participants reported to be employees (full-time: 33%, part-time: 21%, self-employed: 9%), 5% said they were in education and 25% that they were currently at home (stay-at-home: 16%, retired: 9%), with 7% not reporting their current employment status. Participants lived in various regions of the UK, with 31 of 83 UK counties plus London represented in our sample.

The study follows a 2 (salience: parent vs. feminist) × 3 (topic: parent, feminist, identity-neutral) design, with salience as between-subjects factor and topic as a within-subject factor. Participants indicated at the beginning of the study whether they considered themselves to be a parent (yes/no) and/or a feminist (yes/no). Only participants who answered yes to both these questions were included in the sample. Participants were randomized to one of two salient identity conditions: salient identity parent: *n =* 21; salient identity feminist: *n =* 22.

***Materials and procedure.*** Participants were asked to think of themselves as either a feminist or a parent, respectively, depending on the salience condition. They were also asked to write down “up to three things that you and other [feminists/parents] do...” (a) often, (b) rarely, (c) well, and (d) badly (Haslam, Turner, Oakes, McGarty, & Reynolds, 1997). This identity salience manipulation psychologically activates the respective identity by focusing participants on both positive and negative similarities with other group members and the group prototype, without introducing a comparison with, or threat from, a specific out-group (Haslam, 2004).

Every participant was asked to write at least three to five sentences (corresponding to 25 or more words) addressing each of three predefined topics: healthy mealtimes (parent topic), objectification of women (feminist topic), and climate change (identity-neutral topic). The three topics were chosen based on a pretest. In the pretest, *N* = 13 participants (9 women (69%) and 4 men, aged 18–49 years; *M* = 27.77, *SD* = 17.86) rated 26 topics on whether they were typical for a conversation among feminists and parents, respectively. We selected a topic that was perceived to be more typical for feminists than parents (objectification of women: within-participant *t* test, *t*(10) = 4.03, *p* = .002), a topic that was perceived to be more typical for parents than feminists (healthy mealtimes: *t*(11) = 6.20, *p* < .001), and a topic that was perceived as being equally untypical for conversations among parents and feminists (climate change, *t*(11) = 0.00, *p* = 1.00).

In the main study, the audience was held constant across conditions by providing participants at the beginning of the study with information that any texts they wrote would be seen only by the researchers, and not by any other person. No other information about the researchers beyond their university affiliation and name of the lead researcher was provided.

The study was run on the online survey platform LimeSurvey (Schmitz, 2012). Participants were first presented with an information sheet that briefly outlined the study, data protection (including “audience” information), and other ethically relevant information to ensure informed consent. After providing their consent, participants were first asked whether they considered themselves to be a parent/feminist; this was followed by other demographic questions. They were then randomized to one of the two salient identity conditions and received the identity salience manipulation. All participants were asked to write short paragraphs of about five sentences on all three topics. Participants were then debriefed and thanked for their participation.

**Fig. 5:**
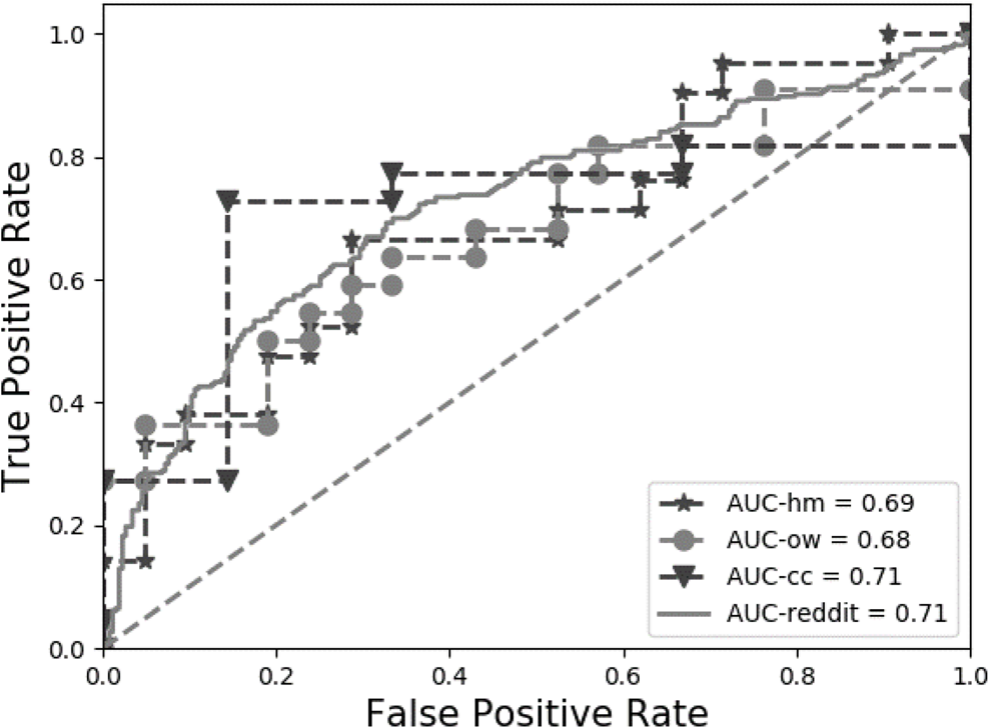
ROC for Studies 3 and 4

***Results.*** We tested the classifier trained on online forum posts of 25 words or more in Study 1 (Mumsnet) on the data from the experimental study. Results show that the model was successful in distinguishing between the two social identities for all three topics, with good predictive accuracy significantly above chance level (see Table 1 and Figs. 2 and 5; for confusion matrices see Supplementary Materials).

**Table 1:** Predictive accuracy for three topics with experimentally manipulated social identity

Topic	AUC	*SE*	Asymptotic 95% *CI*
Identity neutral	.71	.080	.554; .866
Feminist	.68	.082	.518; .841
Parent	.69	.082	.529; .850

Overall, the experiment shows that our model trained on online forum data is valid under experimentally controlled conditions: The model is able to correctly classify a text as being written when a parent or feminist identity was salient, even when the individual holds both identities. This finding supports the idea that a salient identity can be detected through a particular linguistic style pattern that is prototypical for the social group, and that individuals change their linguistic style in line with the salience of their identity. Importantly, the experiment also shows that style differences between groups are not simply due to differing conversational topics or audiences, as both factors were controlled for in our experiment.

### Step 8: Concurrent validity

Once the model has been trained and validated with regard to its construct validity, we need to test its usefulness for research by examining whether the measure is related to outcomes in a theoretically predictable way. For instance, we can assess the model’s ability to distinguish between groups where one of the target identities is salient, and those where salience might pose a problem. This can be done either in an experiment or with naturally occurring data. Differences in salience might be due to social context factors (e.g., a lack of comparative or normative fit in one case but not the other). Alternatively, the model can be used to distinguish between participant groups where one is thought to have difficulties adopting the target identity when the social context would likely make it salient, and one where no such difficulties are expected. Such cases might be expected in new members to a group, low identifiers or dis-identifiers, or those that believe they do not fulfill the requirements to see themselves as a bona fide group member. The hypothesis and data to test concurrent validity will, of course, depend on the particular social identities that are being assessed and the available models that suggest a relationship between the salience of a specific identity and a relevant outcome.

#### Proof of concept: Study 5 – Concurrent validity

In order to test concurrent validity, we chose a sample where one group of participants is expected to have difficulty thinking about themselves in terms of a parent identity, in a situation where parent identity salience is likely to be high. These data also allow us to test whether our model can distinguish between high and low salience in a natural, rather than experimental, context. More specifically, we tested our model on online forum posts in a parenting forum written by primiparous mothers who indicated postnatal mental health difficulties (e.g., depression and/or anxiety) and those who did not indicate such difficulties. Studies show that perinatal depression is prevalent in 9–19% of mothers (and also affects around 10% of fathers; Carlberg, Edhborg, & Lindberg, 2018; Woody, Ferrari, Siskind, Whiteford, & Harris, 2017), with an onset usually within the first three months after birth. Research suggests that maternal role attainment and identification with a parent identity is lower in mothers with perinatal depression (Fowles, 1998; Seymour-Smith et al., 2017). We therefore hypothesized that primiparous mothers with postnatal mental health difficulties would have a lower parent identity salience than those without postnatal mental health difficulties in a context where a parent identity was likely made salient by the social context (here: a parenting forum).

To test this hypothesis, we used posts from the parenting forum Netmums UK (www.netmums.com/coffeehouse). Netmums is a competitor platform to Mumsnet UK that offers a moderated sub-forum for postnatal depression. We received kind permission from Netmums UK to use *N* = 11,497 posts from a forum related to parenting questions after birth, written by *N* = 298 users who had indicated that they were primiparous mothers and had indicated the date of birth or due date in one of their posts. To be included, participants needed to be active forum members during pregnancy and have data for at least one time point between birth and three months after birth. The first data point did not need to be in the month of birth but could be at a later point as long as the month of birth could be identified from a post. Next, we used the unique user ID to identify those mothers in our dataset who had posted in the Netmums postnatal depression forum. Mothers who indicated that they had experienced symptoms of postnatal depression or anxiety, had received a diagnosis of postnatal depression/anxiety, or mentioned medication they were taking for postnatal depression/anxiety were included in the “postnatal mental health difficulties” group (PND group; *N* = 51, 17%). In contrast, mothers who had not posted such information in the postnatal depression forum were included in the “no known postnatal mental health difficulties” group (no PND group; *N =* 247).

Posts in sub-forums related to pregnancy (e.g., Pregnancy Stories) or babies (e.g., Babies (Birth – 12 Months)) were aggregated for each mother for four time points: month of birth/due date (*T*1), one month after birth (*T*2), two months after birth (*T*3), and three months after birth (*T*4). Table 2 shows how many of the primiparous mothers were included at each time point. Although fewer participants are included towards later time points, the two groups had fairly similar rates over time.

**Table 2:** Number of participants for PND and no PND groups across four time points

Groups	*T*1	*T*2	*T*3	*T*4
PND (max *N* = 51)	47 (92%)	34 (67%)	30 (59%)	29 (57%)
No PND (max *N* = 247)	233 (94%)	168 (68%)	157 (64%)	144 (58%)

**Fig. 6:**
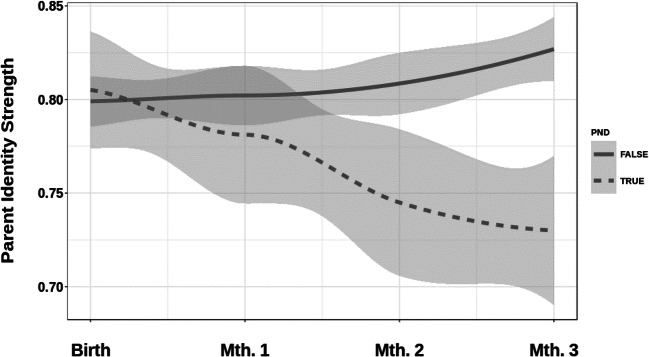
Parent identity salience after birth to three months postnatally for primiparous mothers with postnatal mental health difficulties (PND true) and those who do not report such difficulties (PND false); gray shading indicates uncertainty in the estimate

Using the model trained in Study 1 (Mumsnet), a probability score for having a parent identity salient was calculated based on aggregated posts written during each month. As Fig. 6 shows, parent identity salience is initially high amongst both groups of mothers.

However, compared to the no PND group, the average parent identity salience score declines over time for the PND group, opening up a significant difference three months after the birth of the child (Welch’s *t*(33.12) = 2.07, *p* = .046, Cohen’s *d* = 0.47).

From our results, we can see that primiparous mothers with postnatal mental health difficulties show significantly lower levels of parent identity salience three months after birth than mothers without such difficulties. The results are broadly in line with findings in the literature on postnatal mental health that suggest that postnatal mental health difficulties appear around three months after birth, and are associated with difficulties in maternal role attainment and parental identification (Fowles, 1998; Seymour-Smith et al., 2017).

It needs to be noted here that our model is a relative measure, and effects may be equally due to changes in feminist identity salience. However, all posts were classified as being more likely written with a parent rather than feminist identity salient (Pr > .50), and we have no theoretical reason to believe that PND is associated with an increase in feminist identity. However, our data do not provide conclusive evidence in this regard. The binary nature of our classifier means that we cannot exclude an increase in feminist identity salience, rather than a drop in parent identity salience (or a combination of both), as the explanation for the statistically significant difference between the two groups at *T*4.

Nevertheless, the study provides first evidence for concurrent validity of our measure, and demonstrates its usefulness in analyzing naturally occurring longitudinal data in an applied context. The study also speaks to the construct validity of the method with natural data, as the method can distinguish between posts written by those who are expected to have a parent identity salient (no PND group) and those who are expected to struggle in this regard (PND group), despite writing about the same topics in the same sub-forums. Notably, all subjects in the study were parents, and the posts on which salience was assessed stem from the same forums—which did not include the postnatal depression forum. The latter was only used to assess self-reported mental health difficulties to assign mothers to the two groups. This suggests that the method is, indeed, assessing identity salience, rather than simply a parent identity per se or topic. Furthermore, the finding that all mothers showed equally high probability of parent identity salience in the month of the birth suggests that the method is sensitive to changes within individuals.

### Usage and interpretability

It is important for the correct usage and interpretation of data to reflect on the contexts in which the model can be used. In particular, the binary nature of the classification model imposes restrictions on the research questions for which it can be used. Our general recommendation is to use it as a *research tool and not as a diagnostic tool*. As with other research scales, the exact value for a particular person or post should not be interpreted. Instead, the method can be used to assess changes over time, correlations with relevant outcome variables, or differences between groups/conditions. Furthermore, the binary classifier does not allow for a meaningful interpretation in the following contexts:

- (1) *Neither identity is present/dominant*: The binary classifier provides a continuum between the two identities for which it is trained. It can therefore not be used in a situation where a third identity is dominant in the sample, because the salience of this identity cannot be placed on the continuum of values provided. In our case of a parent/feminist salience classifier, the model scores are ordered on a continuum from “highly likely feminist identity is salient” to “highly likely parent identity is salient”. Applying this classifier to a forum where neither parents nor feminists are the dominant group (e.g., an academic discussion forum where an academic identity is likely to be dominant) does not yield interpretable results between these two poles but will result in misclassification.
- (2) *Mixed identity forums***:** Any categorization of third identities for which the model has not been trained results in misclassification, thereby increasing measurement error. It is therefore important for researchers to understand whether the data would likely lead to a high number of misclassifications. For instance, a news forum that makes different identities salient—depending on the particular news story—is unlikely to be suitable for training or application. Although in naturally occurring contexts it is likely that some posts are written with a different identity salient than those trained, this should not pose a problem as long as at least one of the trained identities is clearly dominant in the dataset.
- (2) *Crossed-categorization:* The binary nature of the classifier means that the midpoint of the scale is ambiguous—it may indicate that both identities are equally salient or that neither identity is salient. Researchers may therefore need to be cautious in how they interpret such a finding. In line with our recommendation above, specific values on the scale should not be interpreted in an absolute sense.

## General discussion

The possibility of automatically inferring the salience of particular social identities from written text promises to shed new light on the context-dependent nature of social identities, their dynamic interplay over extended periods of time, the factors that affect the cognitive accessibility of particular social identities, and the role that linguistic style plays in the expression of social identities. Social identity research has a strong tradition of placing the experiences of the individual within the larger social, cultural, and historical context (Tajfel, 1972; Reicher, 2004). Providing a means by which the study of social identity salience can be taken out of the laboratory and applied in a standardized, easy-to-use way to different types of written texts—from social media, diaries, historical documents, newspapers, and other sources—is therefore of particular importance. To that end, we have introduced ASIA, a method for the construction and validation of a model that automatically assesses the relative salience of one particular identity over another from the linguistic style of a relatively short written text. Thereby, salience can be assessed in real-world contexts without problems incurred by self-report measures such as introspection difficulties, reactivity, social desirability, and other response biases. By also providing a step-by-step open-source tutorial, ASIA can be used to train models for the classification of numerous social identities for which adequate training data can be found, and sets best practice standards for testing the quality of such classifiers. We have placed a particular emphasis on testing the quality of measurement against alternative explanations, a practice that is well-established in psychology but perhaps less emphasized in computational social sciences.

Our example model of feminist and parent identities provides a proof-of-concept case for computational linguistic tools to detect salient social identities as well as shifts between different identities within the same person. We have shown with this example that the assessment of salience in written text can be conducted across different platforms, irrespective of topic or audience, and is not driven by demographic or other stable differences between social groups or local accommodation/linguistic alignment. This gives social scientists the means to study the effects of salient social identities at scale using naturally occurring data and to learn more about the development and impact of social identities in natural social contexts in applied areas such as organizations, healthcare, or education.

Given the ubiquity of group processes in our lives, and their effects on our cognition, emotion, behavior, health, and well-being, we foresee a multitude of research areas that may profit from using ASIA. In particular, ASIA provides an opportunity to test models that theorize changes in the salience of different social identities over time, such as the Social Identity Model of Recovery (SIMOR; Best et al., 2016) with naturally occurring data (see also Best, Bliuc, Iqbal, Upton, & Hodgkins, 2018). In an organizational merger context, for example, a model trained to assess the salience of the “old” versus “new” organizational identity may be used to better understand which factors help employees to acquire the new identity, and how situational factors (e.g., a meeting between employees of the two merged organizations vs. a meeting with a customer) impact on the relative salience of “old” and “new” identities. Similarly, it may be used to understand how the relative salience of subgroup (e.g., ethnic) and superordinate group (e.g., national group) identities varies in different contexts, or between different groups (e.g., first- vs. second-generation immigrants).

By making online data accessible to social psychologists, it can also provide new insights into factors that affect the salience of online identities and test predictions regarding identity recognition and identity performance made by the Social Identity Model of Deindividuation Effects (SIDE; Spears, 2017; Klein et al., 2007). To this end, it may also benefit sociolinguists by providing an additional means by which to study group prototypical linguistic styles. For instance, questions regarding the way new members acquire a group prototypical style, or how a group prototypical style is maintained in the face of majority group pressures, may be examined with the help of an ASIA model.

More generally, ASIA may provide a means by which to examine group prototypes, providing insights into tight and loose norms (Gelfand, 2012; Gelfand, Harrington, & Jackson, 2017), the development of group prototypes over time, and factors that shape the group prototype (Smith, Thomas, & McGarty, 2015). For instance, by looking at changes in the prototypical linguistic style of groups, it may be possible to test to what extent leaders shape the prototype of the group, and to what extent individuals become leaders because they show a better fit with a changed group prototype (Bartel & Wiesenfeld, 2013; Reicher, Haslam, & Hopkins, 2005). Similarly, it may be possible to better understand the dynamics of polarization and fractionalization in intergroup conflict (Esteban & Ray, 2008). As we have recently demonstrated, by combining ASIA with other computational methods such as social network analysis, social influence in online groups can be studied from a social identity perspective (Cork, Everson, Levine, & Koschate, 2020; Turner, 1991).

A further advantage of ASIA is that changes in salience within a person can be studied. The notion that the social context makes a particular social identity salient implies that individuals switch between different identities (e.g., Xiao & van Bavel, 2019), mostly as part of an automatic process. However, crossed-categorization research suggests that it may be possible to have more than one identity salient. In the absence of a method to assess the salience of different groups within an individual, little research is currently available that tests these fundamental questions of identity switching.

### Areas for future development

Although ASIA opens up the possibility of assessing a multitude of salient social identities, it is currently somewhat limited by its binary nature of classifying two different social groups, thereby providing only a relative indicator of salient identity rather than an absolute assessment. A future development of our method is to find a way to assess a single salient identity. However, such a method would need to overcome a theoretical hurdle: the assertion by SCT that the group prototype is context-dependent and relative in nature—it shifts with the comparative context (David & Turner, 1999; Turner et al., 1994). For instance, the prototype of a conservative political party is likely to be further to the right of the political spectrum when in debate with a liberal political party. When in debate with a more right-wing political party, the prototype is likely to shift momentarily towards the political left (Haslam, 2004). Therefore, the linguistic cues to detect a social identity (e.g., feminist) are likely to depend on the relative comparison context (e.g., with parents). If compared with a different social identity (e.g., academic), particular linguistic cues (e.g., long words) may become less predictive. This limits the extent to which several social identities can be part of the same linguistic analysis simultaneously and whether a single identity can be assessed in an absolute sense. For instance, training one social identity against a large number of other identities requires the prototypical style of the social identity to have a unique pattern, rather than being merely distinctive in some style indicators from one other identity. For instance, it may be argued that the feminist prototype is more intellectual than the parent prototype. As a result, a formal/intellectual writing style differentiates a salient feminist identity from a salient parent identity, where an informal/inclusive style is used. However, neither formality, intellectuality, nor inclusivity is an exclusive domain of feminists or parents. Contrasting more groups with a particular group of interest reduces the extent to which distinguishing features can be found, assuming that a group has a “unique” style. Even if such a unique pattern exists, it would likely need a substantially larger amount of written text than the binary classifier due to the finer-grained nature of the classification task.

Follow-up research should also investigate whether ASIA can be used to assess salience in speech in addition to writing. This would allow for the use of a standardized method to assess salience in data from qualitative studies (e.g., interviews, focus groups) and recordings (e.g., of a therapy session). Although style differs between oral and written text (Biber, 1991), it is possible that some of the relatively broad style indicators are used more in one group identity than another. While the absolute number may reduce, the relative frequency between the groups may persist. For instance, speaking in a feminist identity might still lead individuals to use more long words, negative emotion words, and so on than when they speak in a parent identity, even though the overall number of long words, negative emotion words, etc., may be reduced.

In this context, it needs to be noted that the use of several indicators of style as a group-prototypical speech pattern is likely to make the classification more robust against confounds such as a change in platform, topic, or audience, as demonstrated in our proof-of-concept case. Using a single indicator (e.g., we, us) to indicate the salience of an identity is highly vulnerable to factors unrelated to the construct that is being measured and may relate to a multitude of different groups. The use of “bag of words” approaches, such as LIWC, helps to reduce the overreliance on single words, but even here it is important to recognize that a classifier is based on a pattern of several indicators, not a single feature. The presence of a single indicator such as “long words” does not indicate a salient feminist identity, nor does the absence of “long words” indicate the absence of a salient feminist identity.

Importantly, ASIA does not assess whether or not a person has an identity, but only the probability that a particular identity is salient in a specific situation. Follow-up research needs to examine the extent to which the salience of an identity is related to self- (and other-) reports of prototypicality and social identification. Although both of these constructs are distinct from social identity salience, they clearly play a role in the extent to which a salient identity is expressed (McGarty, 2001; van Dick et al., 2011). In fact, our method builds on the theoretical assumption that the salience of an identity will increase prototypical behavior in group members who identify with the respective group. It would therefore be informative to know in which social contexts ASIA may be used as a measure of prototypicality or social identification rather than social identity salience. More work is needed to disentangle these constructs and provide a theoretical model for their relationship with each other and the social context.

In addition, future work should consider the possibility of deception, and whether knowledge of the group prototype is sufficient to successfully mislead an automatic assessment of salient identities. Alternatively, the very act of deception may increase the salience of the actual identity, which should undermine attempts at faking an out-group identity. For instance, Rashid et al. (2013) found higher (rather than equal or lower) success rates of identifying the true demographic categories in an experiment where individuals were asked to fake their age and gender.

## Conclusion

Making naturally occurring data accessible to social psychologists and others interested in social identities allows for investigations into dynamic social identity processes embedded in real-world social and historical contexts. To this end, we have developed ASIA, an analytical protocol for the creation of models that automatically assess the relative salience of two specific social identities in written text. By providing an open-source tutorial and proof-of-concept example on how to construct a model and, importantly, evaluate its quality as a measure, we are equipping researchers with a novel way to assess social identity salience in individuals outside the laboratory. ASIA opens up an opportunity to pursue reproducible analyses of salience effects that can bridge data sources and research traditions, such as computational social sciences, qualitative research, and laboratory experiments. As with all new methods, future work will establish in which contexts ASIA provides a valid assessment of identity salience, and where its limitations lie.

## Supplementary Information

ESM 1 (DOCX 283 kb)

## Data Availability

In the interest of open science and replicability, we provide an accessible step-by-step tutorial of how to replicate our proof-of-concept studies, which can be found on ASIA’s GitHub page (https://github.com/Identity-lab/Tutorial-on-salient-social-Identity-detection-model). The tutorial contains the Python code for preparing the datasets, and the code for training and testing the models. Data for each study, including the necessary LIWC vectors and other relevant variables for each dataset, can be found on OSF: https://osf.io/87t6h/?view_only=a1b5afe488db4014b3f21ed808bcceb9. For ethical reasons (see Step 1), the original posts cannot be shared publicly but are available upon reasonable request from the first author.
