# Supplementary material for: ASIA: Automated Social Identity Assessment using linguistic style
Source: Behav Res Methods. 2021 Feb 11;53(4):1762–81. doi: 10.3758/s13428-020-01511-3 (PMC8367904; doi:10.3758/s13428-020-01511-3)
Supplement: Supplementary file 1 — (DOCX 283 kb) [file 13428_2020_1511_MOESM1_ESM.docx]

**Supplementary Materials**

**1. Word frequencies**

For illustration purposes, we present word frequencies for the most frequent *words* for the five most predictive LIWC style indicators for each social identity based on 50,000 randomly drawn posts from each forum in our Study 1 dataset; importantly, individual style indicators should not be interpreted outside the overall pattern or in an absolute rather than relative way; it also needs to be noted that the ASIA model includes indicators based on punctuation (see Figure 3 in the manuscript), and that indicators in our model are based on normalized frequencies rather than the absolute frequencies presented here for illustration.

***Feminist identity salience***


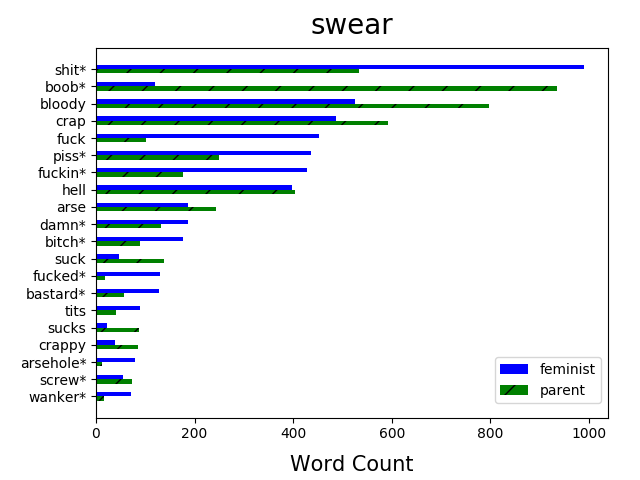

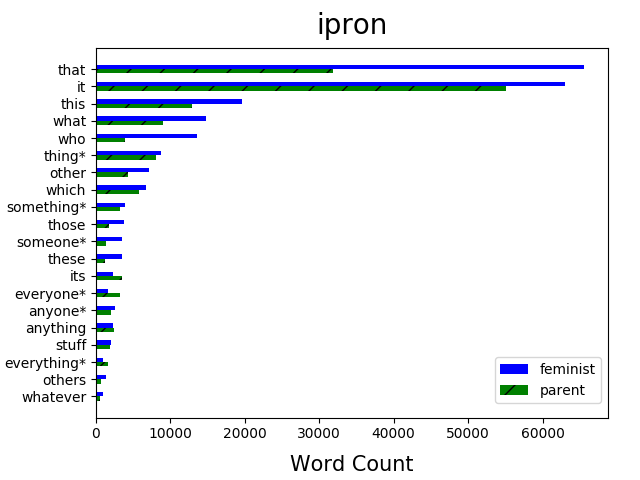

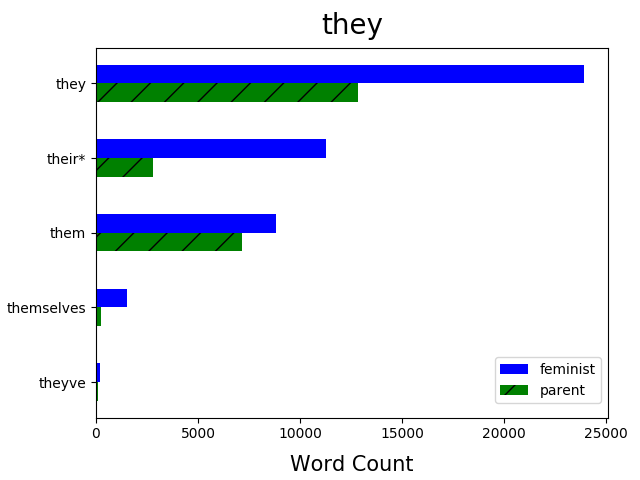

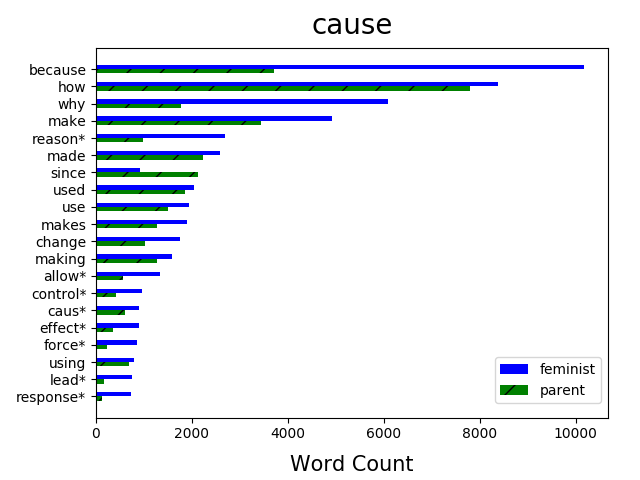

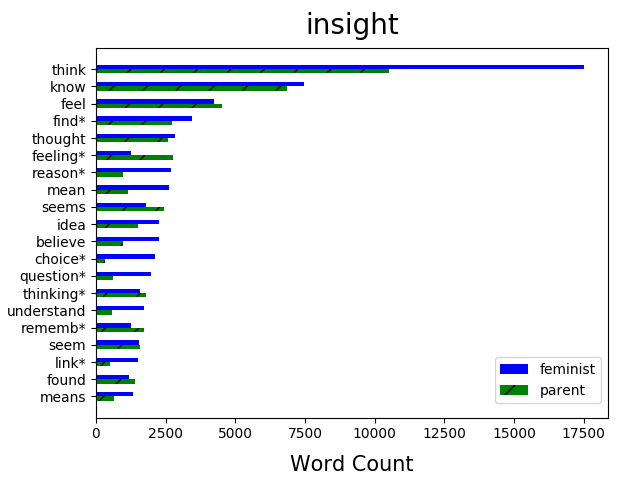


***Parent identity salience***


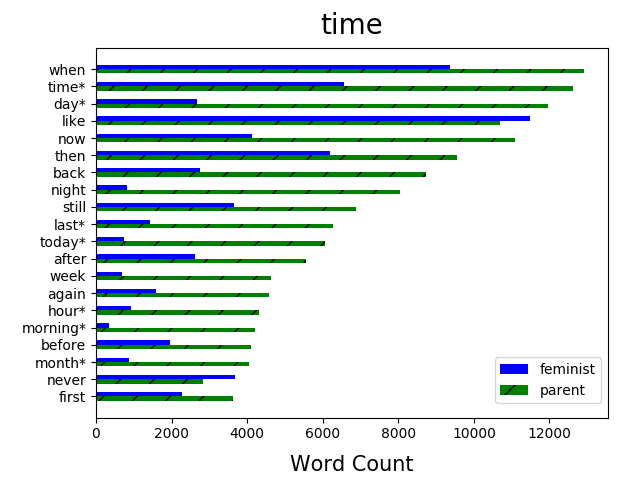

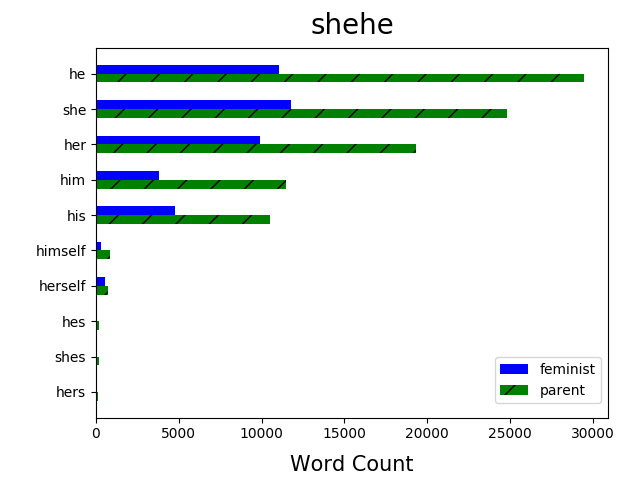


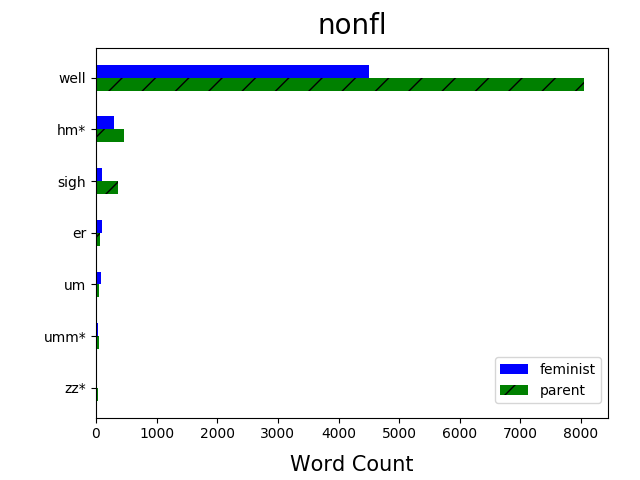

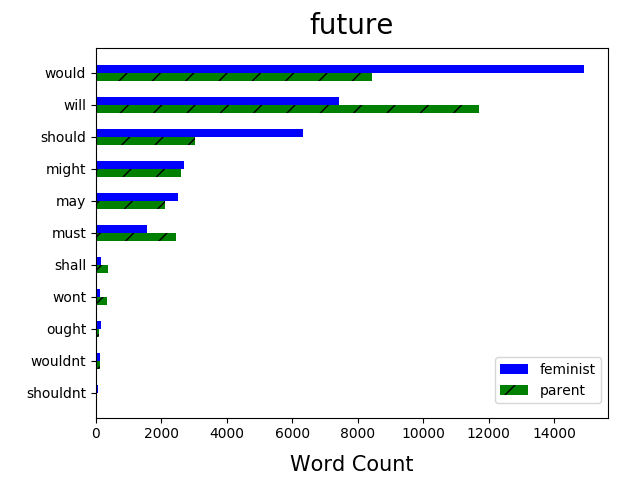


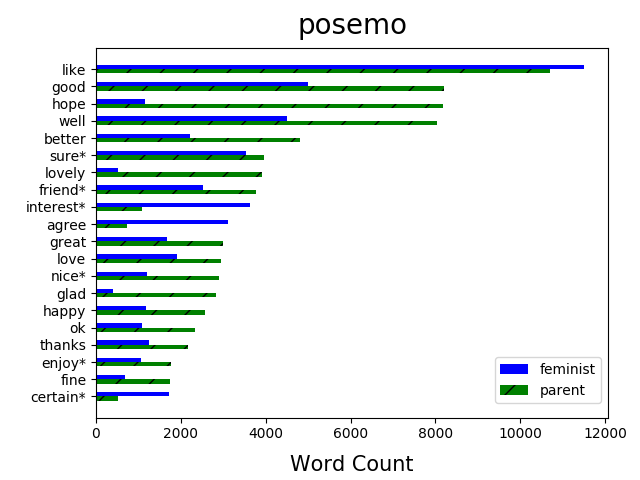


**2. Confusion matrices**

The confusion matrices below show the results when posts are categorised as either feminist or parent based on the resulting probability score being above or below .50; Accuracy (acc) is calculated as the number of correctly classified posts divided by the total number of posts.

As the initial model was trained on data from a different platform (domain) than it is applied to in Studies 3 and 4, even relatively small differences in word frequencies between platforms can result in misclassification – to adjust for domain differences, we use ‘domain adaptation’ (see tutorial [https://github.com/Identity-lab/Tutorial-on-salient-social-Identity-detection-model](https://github.com/Identity-lab/Tutorial-on-salient-social-Identity-detection-model%20) for more details).

***Study 3 (Reddit data)***

| *Before domain adaptation Acc = 60%* | | | |  | *After domain adaptation: Acc = 68%* | | | |
| --- | --- | --- | --- | --- | --- | --- | --- | --- |
| Total  *N =* 526 |  | **Predicted class** | |  | Total  *N* = 526 |  | **Predicted class** | |
| **Actual**  **class** |  | Feminist | Parent |  | **Actual**  **class** |  | Feminist | Parent |
|  | Feminist | 211  (40%) | 52  (10%) |  |  | Feminist | 183  (35%) | 87  (17%) |
|  | Parent | 158  (30%) | 105  (20%) |  |  | Parent | 80  (15%) | 176  (33%) |

***Study 4 (Experimental data)***

***(1) Identity irrelevant topic (climate change)***

| *Before domain adaptation Acc = 58%* | | | |  | *After domain adaptation: Acc = 72%* | | | |
| --- | --- | --- | --- | --- | --- | --- | --- | --- |
| Total  *N =* 43 |  | **Predicted class** | |  | Total  *N* = 43 |  | **Predicted class** | |
| **Actual**  **class** |  | Feminist | Parent |  | **Actual**  **class** |  | Feminist | Parent |
|  | Feminist | 20  (47%) | 2  (5%) |  |  | Feminist | 16  (37%) | 6  (14%) |
|  | Parent | 16  (37%) | 5  (12%) |  |  | Parent | 6  (14%) | 15  (35%) |

***(2) Feminist topic (objectification of women)***

| *Before domain adaptation Acc = 51%* | | | |  | *After domain adaptation: Acc = 63%* | | | |
| --- | --- | --- | --- | --- | --- | --- | --- | --- |
| Total  *N =* 43 |  | **Predicted class** | |  | Total  *N* = 43 |  | **Predicted class** | |
| **Actual**  **class** |  | Feminist | Parent |  | **Actual**  **class** |  | Feminist | Parent |
|  | Feminist | 19  (44%) | 3  (7%) |  |  | Feminist | 13  (30%) | 9  (21%) |
|  | Parent | 18  (42%) | 3  (7%) |  |  | Parent | 7  (16%) | 14  (33%) |

***(3) Parent topic (healthy mealtimes)***

| *Before domain adaptation Acc = 64%* | | | |  | *After domain adaptation: Acc = 62%* | | | |
| --- | --- | --- | --- | --- | --- | --- | --- | --- |
| Total  *N =* 42 |  | **Predicted class** | |  | Total  *N* = 42 |  | **Predicted class** | |
| **Actual**  **class** |  | Feminist | Parent |  | **Actual**  **class** |  | Feminist | Parent |
|  | Feminist | 19  (45) | 2  (5%) |  |  | Feminist | 7  (17%) | 14  (33%) |
|  | Parent | 13  (31%) | 8  (19%) |  |  | Parent | 2  (5%) | 19  (45%) |
